# Supplementary material for: Perceived racial discrimination, resilience, and oral health behaviours of adolescents with immigrant backgrounds
Source: PLoS One. 2025 Jan 3;20(1):e0313393. doi: 10.1371/journal.pone.0313393 (PMC11698319; doi:10.1371/journal.pone.0313393)
Supplement: S1 Table — (DOCX) [file pone.0313393.s001.docx]

**Part 1: Demographic Data (Adolescents)**

1. Date of birth: ____/____/_______ (mm/dd/yyyy)
2. Which grade are you in? Grade ______
3. Sex assigned at birth: □ Male □ Female □ intersex □ prefer not to disclose
4. Were you born in Canada? □ Yes □ No
5. When did your family arrive in Canada? _____________ (YEAR) □ I don’t know
6. What is your family race/ethnicity background? _______________________
7. Are you living with? □ Both parents □ Single parents □ Other, specify ___________
8. What is your mother’s education level?

□ Less than high school □ High school □ College/University □ I don’t know

1. What is your father’s education level?

□ Less than high school □ High school □ College/University □ I don’t know

1. Do you have a dental coverage? □ Yes □ No □ I don’t know

**Part 2: Oral Health Behaviors**

1. When was the last time you visited a dentist or dental hygienist?

□ Within the last 12 months □ Over one year □ Never had one

1. If you had a dental visit, what was (were) the reason(s)? (Check all that apply)

□ Regular check-up □ Non-urgent dental problems □ Urgent dental problems

□ Others (please specify) ______________

1. How many times a day do you brush your teeth?

□ Less than once a day □ Once □ Twice □ More than twice

1. How often do you consume foods or drinks (for example juice, pop, candies, cookies, etc.) high in sugar?

□ Never □ Less often than everyday □ Once a day □ Twice day

□ Three times a day or more often

1. Do you smoke? □ Yes □ No

**Part 3: Adolescent Discrimination Distress Index**

| **Number** | **Questions** | **Have you experienced this because of race or ethnicity**? | **If you had experienced this, did it upset you?**  Not at all Slightly Moderately Considerably Extremely | | | | |
| --- | --- | --- | --- | --- | --- | --- | --- |
| 1 | You were discouraged from joining  an advanced-level class. | Yes / No | 1 | 2 | 3 | 4 | 5 |
| 2 | You were wrongly disciplined or given after-school detention. | Yes / No | 1 | 2 | 3 | 4 | 5 |
| 3 | You were given a lower grade than you deserved. | Yes / No | 1 | 2 | 3 | 4 | 5 |
| 4 | You were discouraged from joining a club. | Yes / No | 1 | 2 | 3 | 4 | 5 |
| 5 | Others your age did not include you in their activities. | Yes / No | 1 | 2 | 3 | 4 | 5 |
| 6 | People expected more of you than they expected of others your age. | Yes / No | 1 | 2 | 3 | 4 | 5 |
| 7 | People expected less of you than they expected of others your age. | Yes / No | 1 | 2 | 3 | 4 | 5 |
| 8 | People assumed your English was poor. | Yes / No | 1 | 2 | 3 | 4 | 5 |
| 9 | You were hassled by police. | Yes / No | 1 | 2 | 3 | 4 | 5 |
| 10 | You were hassled by a store clerk or store guard. | Yes / No | 1 | 2 | 3 | 4 | 5 |
| 11 | You were called racially insulting names. | Yes / No | 1 | 2 | 3 | 4 | 5 |
| 12 | You received poor service at a restaurant or store. | Yes / No | 1 | 2 | 3 | 4 | 5 |
| 13 | People acted as if they thought you were not smart. | Yes / No | 1 | 2 | 3 | 4 | 5 |
| 14 | People acted as if they were afraid of you. | Yes / No | 1 | 2 | 3 | 4 | 5 |
| 15 | You were threatened | Yes / No | 1 | 2 | 3 | 4 | 5 |

**Part 4: BRIEF RESILIENCE SCALR (BRS)**

| **Please respond to each item by marking one box per row** | | **Strongly Disagree** | **Disagree** | **Neutral** | **Agree** | **Strongly Agree** |
| --- | --- | --- | --- | --- | --- | --- |
| **BRS 1** | I tend to bounce back quickly after hard times | 1 | 2 | 3 | 4 | 5 |
| **BRS 2** | I have a hard time making it through stressful events. | 5 | 4 | 3 | 2 | 1 |
| **BRS 3** | It does not take me long to recover from a stressful event. | 1 | 2 | 3 | 4 | 5 |
| **BRS 4** | It is hard for me to snap back when something bad happens. | 5 | 4 | 3 | 2 | 1 |
| **BRS 5** | I usually come through difficult times with little trouble. | 1 | 2 | 3 | 4 | 5 |
| **BRS 6** | I tend to take a long time to get over set-backs in my life. | 5 | 4 | 3 | 2 | 1 |
