# Supplementary material for: Perceived racial discrimination, resilience, and oral health behaviours of adolescents with immigrant backgrounds
Source: PLoS One. 2025 Jan 3;20(1):e0313393. doi: 10.1371/journal.pone.0313393 (PMC11698319; doi:10.1371/journal.pone.0313393)
Supplement: Attachment — Submitted filename: Response to Reviewers.docx [file pone.0313393.s002.docx]

Response to reviewers’ comments

RE: “Perceived Racial Discrimination, Resilience, and Oral Health Behaviours of Adolescents with Immigrant Backgrounds”

Dear Editor,

I would like to thank you and your reviewers for taking the time to consider our manuscript for

publication in PLoS One Journal. The authors would like to thank the reviewers for providing

their constructive feedback so that we may strengthen our manuscript even further.

Below, you will find our responses for each comment made by the reviewers followed by the

revised text if applicable. We also revised manuscript accordingly. Please let us know if you

need any other changes or clarification.

Sincerely,

Maryam Amin, DMD, MSc, PhD

University of Alberta, School of Dentistry

5-513 Edmonton Clinic Health Academy

11405 - 87 Ave NW

Edmonton, AB, T6G 1C9

Email Address: maryam.amin@ualberta.ca

R 1. Please ensure that your manuscript meets PLOS ONE's style requirements, including those for file naming. The PLOS ONE style templates can be found at

<https://journals.plos.org/plosone/s/file?id=wjVg/PLOSOne_formatting_sample_main_body.pdf> and

<https://journals.plos.org/plosone/s/file?id=ba62/PLOSOne_formatting_sample_title_authors_affiliations.pdf>

**Response**: The manuscript was reviewed to ensure the requirements are met.

2. Please provide additional details regarding participant consent. In the ethics statement in the Methods and online submission information, please ensure that you have specified what type you obtained (for instance, written or verbal, and if verbal, how it was documented and witnessed). If the need for consent was waived by the ethics committee, please include this information.

**Response**: Thank you for the comment. We have added a signed consent statement in the manuscript.

3. For studies involving third-party data, we encourage authors to share any data specific to their analyses that they can legally distribute. PLOS recognizes, however, that authors may be using third-party data they do not have the rights to share. When third-party data cannot be publicly shared, authors must provide all information necessary for interested researchers to apply to gain access to the data. (<https://journals.plos.org/plosone/s/data-availability#loc-acceptable-data-access-restrictions>)

For any third-party data that the authors cannot legally distribute, they should include the following information in their Data Availability Statement upon submission:

a) A description of the data set and the third-party source

b) If applicable, verification of permission to use the data set

c) Confirmation of whether the authors received any special privileges in accessing the data that other researchers would not have

d) All necessary contact information others would need to apply to gain access to the data

**Response**: No third-party data was used.

4. Please review your reference list to ensure that it is complete and correct. If you have cited papers that have been retracted, please include the rationale for doing so in the manuscript text, or remove these references and replace them with relevant current references. Any changes to the reference list should be mentioned in the rebuttal letter that accompanies your revised manuscript. If you need to cite a retracted article, indicate the article’s retracted status in the References list and also include a citation and full reference for the retraction notice.

**Response**: Reference list has been checked.

__________________________________________________________________________

**Reviewer #1: Dear Authors,**
Thank you for your excellent work.
The current study is on a topic of relevance to the scope of the journal. I found the paper to be very interesting and the authors had chosen an important topic in the field of social and behavioral determinants of oral health. well-conducted and well-written paper.

**Response**: Thank you for your comment. No changes needed.

­­­­­­­­­­­­­­­­­­­­­­­­­________________________________________________________________

**Reviewer2:**

I was honored by this review participation. Based on my limited knowledge, I have few points which my aid in highlighting better presentation of this informative research. A huge and intensive effort were evidenced throughout the manuscript, Critical aspect was explored affecting community and wellbeing. The following could be beneficial:

Abstract: Very informative abstract but conclusion is limited and very generalized

**- Response**: Thank you for your comment. We have expanded on the conclusion section in the abstract.

**- Revised text**: Perceived racial discrimination was associated with the pattern of dental attendance, toothbrushing frequency, and self-rated oral health. Resilience did not moderate the observed associations.

Introduction:
- Overall, the introduction is inclusive with holistic view, but more should be added to physical effect of discrimination which is briefly mentioned.

**- Response**: Thank you for your comment. We have expanded on the physical effect of discrimination.

**- Revised text**: The stress associated with experiences of discrimination may trigger physiological responses that contribute to physical health problems such as cardiovascular diseases and obesity (18,19). A systematic review analysed the quantitative relationship between perceived racial discrimination and hypertension, finding a significant association between the two (20). In addition, to cope with discrimination, individuals may engage in unhealthy behaviours as maladaptive coping mechanisms. Studies have reported the association between perceived racial discrimination and unhealthy behaviours like substance abuse (21).

- a repeated statement with different references page (5) lines (128-131):
" and physical health problems like hypertension, obesity, substance abuse, and self-reported poor health (15–17), and physical health problems like hypertension, obesity, substance abuse, and self-reported poor health (18–21)"

**-Response**: Thank you for your comment. The repeated statement has been removed and the reference has also been corrected.

**- Revised text**: Perceived racial discrimination has been correlated with several mental health problems like depression, psychological distress, and anxiety (15-17). The stress associated with experiences of discrimination triggers physiological responses that contribute to physical health problems like cardiovascular diseases, and obesity (18.19)

- For analytical research like this one, it is more suitable to state a hypothesis

**-Response**: Thank you for your comment. A hypothesis has been added at the end of the introduction section.

**- Revised text**: It was hypothesized that the perceived racial discrimination would negatively impact oral health behaviours and resilience would moderate this association.

Methodology:
This section in the manuscript is very short and lacking details.

- There is data mentioned in the abstract is mentioned under the methodology section which should be harmonized:
* Study design is missing in the methodology section while it is mentioned in the abstract as cross-sectional study. It was mentioned in the abstract that " nine community organizations" while number of the organizations is not mentioned in the methodology also the data about the community organization and their locations and coverage area for each (inside Alberta or outside), how they are serving immigrants (Briefly) and how you did such communication, did you communicate other organizations, but they refused or all the organization you communicated with approved to participate.

Convenience sampling (abstract) - snowball (methodology section), besides it should be explained more in the methodology section not to be briefed as mentioning that snowball is one of the ways to perform Convenience sampling.

**-Response**: Thank you for your comment. More details have been added to the methods section.

- Expert opinion for content validity is missing although a validated tools was used but those tools are only part of the questionnaire and the whole questionnaire should go through content validation by field experts.

**-Response**: Thank you for your comment. Our questionnaire consisted of four sections. The first two sections, which collected information on demographics and oral health behaviours, included standard questions that have been widely used in previous studies. ^1,2^ The third and fourth sections, which measured perceived racial discrimination and resilience, were also based on previously validated instruments. ^3,4^ Therefore, the authors did not deem it necessary to conduct additional content validity testing.

- Do participants need to complete the whole questionnaire or the can leave questions without answers (optionally), also can they withdraw at any point.

**-Response**: Thank you for your comment. The participant had the option to withdraw at any point, but they had to answer all the questions in order to minimize the number of missing data.

**- Revised text**: Prior to data collection, signed consent was obtained from parents, along with signed assent from the adolescent participants. The participants were explained that they could withdraw from the study at any time. To ensure accessibility, the questionnaire was available in both print and online formats. The participants were asked to answer all the questions, but had the option “Do not know” in cases they had limited information about the topic of interest.

- Under the " Data Collection and procedures", only questionnaire sections were mentioned without number of questions included under each section, also the template or the stem questions is not attached to the manuscript or even added as appendix.

**-Response**: Thank you for your comment. The number of questions has been added in each section, The questionnaire has been added as appendix.

**- Revised text**: The questionnaire administered to participants included four distinct sections (S1 table). The first section included 10 questions that gathered demographic details about the adolescents and their families. The second section centered on 6 specific oral health behaviours, serving as the study's outcome or dependent variables. The third section assessed perceived racial discrimination using a 15-item validated scale and the fourth section assessed resilience using a 6-item validated scale.Top of FormTop of Form

- Under the " Outcome Variables", only self-rated oral health condition is explained while the others were just mentioned.

**-Response**: Thank you for your comment. An explanation of all the variables has been included.

**- Revised text**: In this study, outcome variables included participants' oral health behaviours and self-rated oral health. Self-rated oral health condition was assessed with a single question asking participants to rate their oral health from "very good" to "good" "fair", "poor", and “not good”. Oral health behaviours were assessed by questions asking about the tooth brushing frequency per day (less than one, once, twice or more), sugar consumption frequency (never or less often than every day, once a day, twice a day, or more often), participant’s use of dental services (within last 12 months, more than a year) the pattern for dental attendance (regular check-up, urgent/non-urgent dental problem), and smoking (yes or no).

Results
The order of the variables in mentioning through the tables is different and also different from the order in the methodology section, this is particularly with oral health outcomes which should be unified.

**-Response**: Thank you for your comment. The order of the variables has been unified in the methodology, result section and tables.

**- Revised text**: Analysis of oral health behaviours revealed that more than half of the participants (57.28%) self-assessed their oral health as good. Around 62% of participants brushed their teeth twice or more daily, and 73.42% consumed high-sugar foods or beverages between main meals at least once a day. Around 45.57% of participants had a dental visit in the past year, with 65.53% of these visits being regular check-ups. The specifics of participants' oral health behaviours are outlined in Table 2.

- Table 1: percentages are missing the % symbol besides the numbers as this sometimes cause confusion while it is added in the other tables. Within the context talking about table 1, it is never mentioned that this table also represents the percentages of the perceived racial discrimination along with the demographic data.

**-Response**: Thank you for your comment. The percentage symbol has been added in Table1. We have also added in the result section that table 1 present demographic data with and without racial discrimination.

**- Revised text**: Chi-square test was conducted to explore statistical proportion differences of demographic variables with and without racial discrimination. A comprehensive overview of participant demographics with and without racial discrimination is presented in Table 1.

- Non-respondents' number and their percentage per variable is not mentioned

**-Response**: Thank you for your comment. Since the participants were told to answer all the questions we do not have non-respondent values to report.

- Modalities that did not receive selection under table 2 is not totally mentioned, as under the Self-rated oral health (fair & Poor) were not included even they are not selected but they are mentioned in the methodology.

**-Response**: Thank you for your comment. The oral health behaviours in our questionnaire had 3 to 4 options, but for analysis, we grouped the categories to get binary variables for instance “not good” and “poor” categories were combined as “not good”. Similarly, “fair” and “good” and “very good” categories were combined as “good”. An explanation of this has been added in the methodology section.

**- Revised text**: For analysis, we merged the categories of the outcome variables and reported them as binary variables.

- Comments on Likert scale results are missing the resultant tendency as this tendency indicative of the severity of distress.

**-Response**: Thank you for your comment. Comments on Likert scale results have been added.

**- Revised text**: Overall, the data for discrimination distress showed that the majority of respondents report low levels of distress (mean values around 1.3-1.5), with moderate variability (standard deviations between 0.47-0.64). While some respondents indicated higher distress (ranges up to 4.40), the overall severity remains low.

Discussion:

- The discussion section missing any justification or comments on the initial descriptive results and almost few about the univariate analysis, while correlations only justified.

**-Response**: Thank you for your comment. A justification of some descriptive results has been included.

**- Revised text**: Our study found that 65.5% of adolescents had dental check-ups in the past year, a similar prevalence of regular check-ups among adolescents has been reported in other research (27,46). About 62% brushed their teeth twice daily, with significant links to living status, dental coverage, and father’s education consistent with prior research indicating a link between adolescents' tooth brushing habits and socio-demographic factors (47,48). Sugar consumption also correlated significantly with living status, dental coverage, and mother’s education, reflecting trends reported in other studies related to parental influence and adolescents' health knowledge (49,50).

- Regarding the important point in descriptive results is the high percentage of perceived discrimination, is it normal to find this percentage and what about reported studies addressing this issue in a matter of existence before talking about correlation with oral health and the efforts spent in management of this serious issues (in brief). A separate section gathering recommendations with proposed actions which should be alerting to the community organizations and even the official authorities while if applicable to share this valuable investigation with them.

**-Response**: Thank you for your comment. A brief explanation about this has been added to the discussion.

**- Revised text**: About three-quarters of the participants reported experiencing some discrimination because of their race. This prevalence aligns closely with findings from other studies concerning perceived racial discrimination among immigrant populations (78,79). Given its significant impact on adolescent health, as evidenced by our findings and supported by various studies, it is crucial for community organizations and authorities to promptly implement proactive measures. The association between racial discrimination and adverse oral health outcomes highlights the broader impact of discrimination on overall well-being. This connection highlights the urgent need for comprehensive strategies that address the root causes of discrimination and its far-reaching effects. Community organizations should lead the charge by launching targeted awareness campaigns that educate the public on the harmful effects of racial discrimination. These campaigns should aim to foster environments that celebrate diversity, promote inclusivity, and actively challenge racial biases. Through education and engagement, these organizations can empower individuals and communities to recognize and combat discrimination in all its forms. Meanwhile, authorities have a critical role in implementing and rigorously enforcing anti-discrimination policies, particularly within schools and healthcare institutions. These policies must ensure that all adolescents, regardless of race or ethnicity, have equitable access to the resources and support they need. This includes creating safe spaces where adolescents can voice their experiences and receive the necessary interventions to mitigate the impact of discrimination on their health.

­­­­­­­­­­­­­­­­­­­­­­­­­­­­__________________________________________________________________________________

**Additional Editor Comments:**

I have a few queries after going through the manuscript.

1. The abstract is started with the objectives of the study.

**-Response**: Thank you for your comment. The start of the abstract has been changed to “INTRODUCTION”

**- Revised text**:

Introduction: Unmet oral health needs remain a significant issue among immigrant adolescents, often exacerbated by experiences of racial discrimination. This study aimed to examine the associations between perceived discrimination and oral health behaviours in adolescents with immigrant backgrounds and explore the potential moderating effect of resilience on this association.

2. In the result section, it is mentioned that ‘average age of 15.3 ± 1.9 years’. What is ± 1.9? Is it a standard deviation? Please clarify.

3. ‘IQR’ full form is missing in the manuscript.

**-Response**: Thank you for your comment. The standard deviation (SD) and IQR full form have been added in the result section.

**- Revised text**: A total of 316 participants were recruited between June 2022 and August 2023 for this study. The participants had a mean age of 15.3 years (SD = 1.9), and a median age of 15 years (Inter Quartile Range: 12-18).

4. Mention the type of correlation determined in Tables 3 and 4. Organise the tables properly.

**-Response**: Thank you for your comment. The type of correlation determined in table 3 and 4 has been added.

**- Revised text**:

Page 15, lines (325-327)

Table 3 outlines the correlation between oral health behaviours and demographic factors. Based on the type of variable, different types of correlation methods were used to assess the correlation of oral health behaviour with demographics (point biserial for continuous variables and Cramer V for categorical variables).

Page 17, lines (368-369)

As shown in Table 4, Point biserial correlation has been reported for oral health behaviours and discrimination score

5. The adjusted odds ratios are calculated in the manuscript. Were all the important confounders considered for analysis? Mention the results of the Goodness of Fit test of the models. Also, mention the values of pseudo-R-squared.

**-Response**: Thank you for your comment. We used the purposeful selection method to determine the potential confounding factors for our regression models. All the models have been reassessed and the given odd ratios have been adjusted for important confounders. The goodness of fit value have been added in the result section. Pseudo-r-squared values has been added at the footnotes of the table 5 and 6

**- Revised text**:

For self-rated oral health model-

Page 19, line (390-392)

In adjusted logistic regression analyses, , the chi-square for the Hosmer and Lemeshow test for the final model was 12.4 and statistically non-significant indicating that the model fit the data reasonably well.

For tooth brushing model-

Page 19, line (396-398)

In adjusted logistic regression analyses, the chi-square for the Hosmer and Lemeshow test for the final model was 3.72 and statistically non-significant indicating that the model fit the data reasonably well.

For pattern for dental attendance model -

Page 19, line (405-408)

Top of Form

In adjusted logistic regression analyses, the chi-square for the Hosmer and Lemeshow test for the final model was 4.77, and statistically non-significant indicating that the model fit the data reasonably well.

Table 5. Adjusted odds ratio of oral health behaviours and discrimination distress score: multivariate analyses.

| Oral health behaviors | Peer discrimination distress  Odds Ratio (95% CI) | Educational discrimination distress  Odds ratio (95% CI) | Institutional discrimination distress  Odds ratio (95% CI) | Total discrimination distress  Odds ratio (95% CI) |
| --- | --- | --- | --- | --- |
| Self-rated oral health*^a^ | 0.67(0.46-0.96) * | 0.67(0.45-1.02) | 0.49(0.29-0.81) * | 0.49(0.29-0.81) * |
| Tooth brushing frequency*^b^ | 0.60(0.42-0.87) * | 0.61(0.41-0.90) * | 0.44(0.26-0.73) * | 0.42(0.25-0.71) * |
| Sugar consumption between meals^c^ | 1.50(1.01-2.50) | 1.30(0.82-2.01) | 1.70(0.89-3.22) | 1.90(0.98-3.41) |
| Utilization of dental services^d^ | 1.10(0.70-1.56) | 0.83(0.57-1.21) | 1.07(0.67-1.69) | 1.01(0.63-1.60) |
| Pattern for dental attendance*^e^ | 1.50(1.03-2.50) * | 1.60(1.06-2.50) * | 2.20(1.30-2.70) * | 2.30(1.30-4.00) * |

*Significant association

^a^ adjusted for coverage (pseudo R^2^=0.05)

^b^ adjusted for father’s education, living status (pseudo R^2^=0.06).

^c^ adjusted for ethnicity, Canada-born and living status (pseudo R^2^=0.06).

^d^ adjusted for ethnicity (pseudo R^2^=0.02).

^e^ adjusted for ethnicity, Canada-born, coverage, and father’s education (pseudo R^2^=0.15).

Table 6. Adjusted Odds ratio of oral health behaviours and resilience: multivariate analysis.

| Oral health behaviors | Odds ratio (95% CI) |
| --- | --- |
| Self-rated oral health*^a^ | 3.30(2.10-5.30) * |
| Tooth brushing frequency*^b^ | 2.20(1.40-3.40) * |
| Sugar consumption between meals^c^ | 0.66(0.41-1.06) |
| Utilization of dental services^d^ | 1.48(0.98-2.20) |
| Pattern for dental attendance*^e^ | 0.42(0.25-0.69) * |

*(p<0.001) Significant association

^a^ adjusted for coverage and living status (pseudo R^2^=0.11).

^b^ adjusted for father’s education and living status (pseudo R^2^=0.06).

^c^ adjusted for ethnicity, Canada-born, and living status (pseudo R^2^=0.06).

^d^ adjusted for ethnicity (pseudo R^2^=0.02).

^e^ adjusted for ethnicity, coverage, and father’s education (pseudo R^2^=0.15).

6. Mention exact p values in Table 6.

**-Response**: Thank you for your comment. Exact p values for all significant association in table 6 is less than 0.001. We had added it at the footnote.

**- Revised text**:

Table 6. Adjusted Odds ratio of oral health behaviours and resilience: multivariate analysis.

| Oral health behaviors | Odds ratio (95% CI) |
| --- | --- |
| Self-rated oral health*^a^ | 3.30(2.10-5.30) * |
| Tooth brushing frequency*^b^ | 2.20(1.40-3.40) * |
| Sugar consumption between meals^c^ | 0.66(0.41-1.06) |
| Utilization of dental services^d^ | 1.48(0.98-2.20) |
| Pattern for dental attendance*^e^ | 0.42(0.25-0.69) * |

*(p<0.001) Significant association

^a^ adjusted for coverage and living status (pseudo R^2^=0.11).

^b^ adjusted for father’s education and living status (pseudo R^2^=0.06).

^c^ adjusted for ethnicity, Canada-born, and living status (pseudo R^2^=0.06).

^d^ adjusted for ethnicity (pseudo R^2^=0.02).

^e^ adjusted for ethnicity, coverage, and father’s education (pseudo R^2^=0.15).

References

1.	Amin, M., Schumacher, C. & Bohlouli, B. Perceived social support and discrimination and oral health behaviors in adolescents. *Clin. Exp. Dent. Res.* **7**, 1183–1189 (2021).

2.	Bohlouli, S., Dolatabadi, S., Bohlouli, B. & Amin, M. Racial discrimination, self-efficacy, and oral health behaviours in adolescents. *PLOS ONE* **18**, e0289783 (2023).

3.	Smith, B. W. *et al.* The brief resilience scale: assessing the ability to bounce back. *Int. J. Behav. Med.* **15**, 194–200 (2008).

4.	Fischer, A. R. & Shaw, C. M. African Americans’ mental health and perceptions of racist discrimination: The moderating effects of racial socialization experiences and self-esteem. *J. Couns. Psychol.* **46**, 395–407 (1999).
