# Supplementary material for: Perceived racial discrimination, resilience, and oral health behaviours of adolescents with immigrant backgrounds
Source: PLoS One. 2025 Jan 3;20(1):e0313393. doi: 10.1371/journal.pone.0313393 (PMC11698319; doi:10.1371/journal.pone.0313393)
Supplement: Attachment — Submitted filename: Response to reviewers 2.docx [file pone.0313393.s003.docx]

Response to reviewers’ comments

RE: “Perceived Racial Discrimination, Resilience, and Oral Health Behaviours of Adolescents with Immigrant Backgrounds”

Dear Editor,

I would like to thank you and your reviewers for taking the time to consider our manuscript for

publication in PLoS One Journal. The authors would like to thank the reviewers for providing

their constructive feedback so that we may strengthen our manuscript even further.

Below, you will find our responses for each comment made by the reviewers followed by the

revised text if applicable. We also revised manuscript accordingly. Please let us know if you

need any other changes or clarification.

Sincerely,

Maryam Amin, DMD, MSc, PhD

University of Alberta, School of Dentistry

5-513 Edmonton Clinic Health Academy

11405 - 87 Ave NW

Edmonton, AB, T6G 1C9

Email Address: maryam.amin@ualberta.ca

Additional Editor Comments:

The author revised the manuscript. However, further revision is required to consider the following points.

1. Mention the pseudo-R-squared used. e.g., Nagelkerke's R², Cox and Snell’s R² etc.

**- Response**: Thank you for your comment. We have mentioned below Tables 5 and 6 the pseudo-R-squared reported is McFadden’s pseudo-R².

**- Revised text**:

Table 5. Adjusted odds ratio of oral health behaviours and discrimination distress score: multivariate analyses.

| Oral health behaviors | Peer discrimination distress  Odds Ratio (95% CI) | Educational discrimination distress  Odds ratio (95% CI) | Institutional discrimination distress  Odds ratio (95% CI) | Total discrimination distress  Odds ratio (95% CI) |
| --- | --- | --- | --- | --- |
| Self-rated oral health*^a^ | 0.67 (0.46-0.96) * | 0.67 (0.45-1.02) | 0.49 (0.29-0.81) * | 0.49 (0.29-0.81) * |
| Tooth brushing frequency*^b^ | 0.60 (0.42-0.87) * | 0.61 (0.41-0.90) * | 0.44 (0.26-0.73) * | 0.42 (0.25-0.71) * |
| Sugar consumption between meals^c^ | 1.50 (1.01-2.50) | 1.30 (0.82-2.01) | 1.70 (0.89-3.22) | 1.90 (0.98-3.41) |
| Utilization of dental services^d^ | 1.10 (0.70-1.56) | 0.83 (0.57-1.21) | 1.07 (0.67-1.69) | 1.01 (0.63-1.60) |
| Pattern for dental attendance*^e^ | 1.50 (1.03-2.50) * | 1.60 (1.06-2.50) * | 2.20 (1.30-2.70) * | 2.30 (1.30-4.00) * |

*Significant association

^a^ adjusted for coverage (McFadden pseudo R^2^=0.05).

^b^ adjusted for father’s education, living status (McFadden pseudo R^2^=0.06).

^c^ adjusted for ethnicity, Canada-born and living status (McFadden pseudo R^2^=0.06).

^d^ adjusted for ethnicity (McFadden pseudo R^2^=0.02).

^e^ adjusted for ethnicity, Canada-born, coverage, and father’s education (McFadden pseudo R^2^=0.15).

Table 6. Adjusted Odds ratio of oral health behaviours and resilience: multivariate analysis.

| Oral health behaviors | Odds ratio (95% CI) |
| --- | --- |
| Self-rated oral health*^a^ | 3.30 (2.10-5.30) * |
| Tooth brushing frequency*^b^ | 2.20 (1.40-3.40) * |
| Sugar consumption between meals^c^ | 0.66 (0.41-1.06) |
| Utilization of dental services^d^ | 1.48 (0.98-2.20) |
| Pattern for dental attendance*^e^ | 0.42 (0.25-0.69) * |

* Significant association

^a^ adjusted for coverage and living status (McFadden pseudo R^2^=0.11) (p<0.001).

^b^ adjusted for father’s education, and living status (McFadden pseudo R^2^=0.06) (p<0.001).

^c^ adjusted for ethnicity, Canada-born and living status (McFadden pseudo R^2^=0.06) (p=0.08).

^d^ adjusted for ethnicity (McFadden pseudo R^2^=0.02) (p=0.26).

^e^ adjusted for ethnicity, coverage, and father’s education (McFadden pseudo R^2^=0.15) (p<0.001).

2. Include the p-value for the Hosmer Lemeshow test and the interpretation of the results thereof.

**- Response**: Thank you for your comment. We have included the p-value for the Hosmer Lemeshow test and their interpretation

**- Revised text**:

For the Oral health model, page 17, lines 364-366
In adjusted logistic regression analyses, the chi-square for the Hosmer and Lemeshow test for the final model was 12.4, and statistically non-significant (p=0.13) indicating that the model fit the data reasonably well.

For the brushing frequency model, page 18, lines 370-373

In adjusted logistic regression analyses, the chi-square for the Hosmer and Lemeshow test for the final model was 3.72, and statistically non-significant (p= 0.81) indicating that the model fit the data reasonably well.

For Pattern of dental visit model, page 18, lines 380-383

In adjusted logistic regression analyses, the chi-square for the Hosmer and Lemeshow test for the final model was 4.77, and statistically non-significant (p=0.78), indicating that the model fit the data reasonably well.

3. The % symbol is repeated in the rows in Tables 1 and 2. There is no need to repeat the % symbol if it is included in the column heading. Organize the tables properly so that they are easily understandable by the reader. Spacing between a point estimate and round brackets is missing, and it is also missing in other tables.

**- Response**: Thank you for your comment. % symbol has been removed in Table 1 and 2. Spacing between the point estimate and round brackets have been checked in all the tables

4. Mention the exact p-values for Table 1. Mention the unit for age in Table 1.

**- Response**: Thank you for your comment. The exact P value has been added in Table 1. Unit of age (years) has also been added.

**- Revised text**:

Table 1. Demographic characteristics of the participants (N=316)

| Characteristics | n (%) | With racial discrimination  (n=241) | Without racial discrimination (n=75) | p-value |
| --- | --- | --- | --- | --- |
| Age(years)-Mean (SD)  Median (Range) | 15.3 (1.9)  15 (12-18) | 15.5 (1.9) | 14.7 (1.9) | 0.002 |
| Gender  Female  Male  Prefer not to disclose | 177 (56.01)  136 (43.04)  3 (0.95) | 136 (56.43)  102 (42.32)  3 (1.24) | 41 (54.67)  34 (45.33)  0 (0.00) | 0.50 |
| Born in Canada  No  Yes | 174 (55.06)  142 (44.94) | 137 (56.85)  104 (43.15) | 37 (49.33)  38 (50.67) | 0.20 |
| Ethnicity  Indian  Filipino  Chinese  Nepali  African  Others | 101 (31.96)  74 (23.42)  48 (15.19)  39 (12.34)  36 (11.39)  18 (5.70) | 74 (30.71)  59 (24.48)  39 (16.18)  22 (9.13)  31 (12.86)  16 (6.64) | 27 (36.00)  15 (20.00)  9 (12.00)  17 (22.67)  5 (6.67)  2 (2.67) | 0.02 |
| Living status  Both parents  Single  Others | 268 (84.81)  39 (12.34)  9 (2.85) | 200 (82.99)  33 (13.69)  8 (3.32) | 68 (90.67)  6 (8.00)  1 (1.33) | 0.29 |
| Father’s education  High school /less  College/university Don’t know | 52 (17.09)  216 (71.52)  36 (11.39) | 47 (19.50)  165 (68.46)  29 (12.03) | 7 (9.34)  61 (81.33)  7 (9.33) | 0.07 |
| Mother’s education  High school /less  College/university  Don’t know | 66 (20.89)  230 (72.78)  20 (6.33) | 53 (21.99)  172 (71.37)  16 (6.64) | 13 (17.34)  58 (77.33)  4 (5.33) | 0.58 |
| Dental Coverage  Yes  No  Don’t know | 199 (62.97)  97 (30.70)  20 (6.33) | 149 (61.83)  81 (33.61)  11 (4.56) | 50 (66.67)  16 (21.33)  9 (12.00) | 0.02 |

5. Table 6 shows that the three independent variables' p-values are <0.001. p-values for the other two variables are missing.

**- Response**: Thank you for your comment. The p-values for the other two variables have been added.

**- Revised text-**

Table 6. Adjusted Odds ratio of oral health behaviours and resilience: multivariate analysis.

| Oral health behaviors | Odds ratio (95% CI) |
| --- | --- |
| Self-rated oral health*^a^ | 3.30 (2.10-5.30) * |
| Tooth brushing frequency*^b^ | 2.20 (1.40-3.40) * |
| Sugar consumption between meals^c^ | 0.66 (0.41-1.06) |
| Utilization of dental services^d^ | 1.48 (0.98-2.20) |
| Pattern for dental attendance*^e^ | 0.42 (0.25-0.69) * |

* Significant association

^a^ adjusted for coverage and living status (McFadden pseudo R^2^=0.11) (p<0.001).

^b^ adjusted for father’s education, and living status (McFadden pseudo R^2^=0.06) (p<0.001).

^c^ adjusted for ethnicity, Canada-born and living status (McFadden pseudo R^2^=0.06) (p=0.08).

^d^ adjusted for ethnicity (McFadden pseudo R^2^=0.02) (p=0.26).

^e^ adjusted for ethnicity, coverage, and father’s education (McFadden pseudo R^2^=0.15) (p<0.001).

6. In Table No. 2, under the variable ‘pattern for dental attendance’, the total is 293. Recheck once again.

**- Response**: Thank you for your comment. The remaining participants (21) were not included in the “pattern for dental attendance” as they had never visited a dentist. However, we have explained in the result section and also listed the number in Table 2.

**- Revised text**: Pattern of dental visits was reported by 293 participants with 65.53%of these visits being regular check-ups. The remaining 23 participant reported that they had never visited a dentist

Table 2. Dependent and independent variables (N=316).

| VARIABLES | n (%) |
| --- | --- |
| Self-rated oral health  Good  Not Good | 181 (57.28)  135 (42.72) |
| Toothbrushing frequency (per day)  Once or Less  Twice or More | 120 (37.97)  196 (62.03) |
| Sugar consumption between meals  Never or less than every day  Once a day or more | 84 (26.58)  232 (73.42) |
| Utilization of dental services (last year)  Within 12 months  Over one year | 144 (45.57)  172 (54.43) |
| Pattern for dental attendance (n=293)  Regular check-up  Dental problem | 192 (65.53)  101 (34.47) |
| Smoke  No  Yes | 307 (97.15)  9 (2.85) |
| Prevalence of Racial discrimination  Yes  No | 241 (76.27)  75 (23.73) |
| Total Discrimination Distress score  Peer Discrimination Distress Score  Educational Discrimination Distress Score  Institutional Discrimination Distress Score  Resilience | M[SD][Range]  1.40 (0.47) (1.00-3.40)  1.49 (0.64) (1.00-4.20)  1.42 (0.60) (1.00-4.40)  1.31 (0.49) (1.00-4.00)  3.10 (0.55) (1.50-4.30) |
